# High Resistance to Azithromycin in Clinical Samples from Patients with Sexually Transmitted Diseases in Guangxi Zhuang Autonomous Region, China

**DOI:** 10.1371/journal.pone.0159787

**Published:** 2016-07-28

**Authors:** Bangyong Zhu, Jin Bu, Wei Li, Jie Zhang, Geng Huang, Juan Cao, Zhongshu Tang, Quan Gan, Pingjiang Wei

**Affiliations:** 1 Central laboratory, Guangxi Institute of Dermatology, Nanning, Guangxi Zhuang Autonomous Region, China; 2 Editorial Department of Journals of Nanjing Medical University, Nanjing Medical University, Nanjing, Jiangsu, China; 3 Department of Dermatology, Guangxi Institute of Dermatology, Nanning, Guangxi Zhuang Autonomous Region, China; Imperial College London, UNITED KINGDOM

## Abstract

Azithromycin is used as an alternative medicine in patients with syphilis who are intolerant to penicillin. Nevertheless, the report of treatment failure of azithromycin for patients with syphilis has raised concerns in China in the past years. In this study, 178 patients with early syphilis, who were treated in sexually transmitted infections clinics in four cities in Guangxi Zhuang Autonomous Region were enrolled to investigate the regional prevalence of *Treponema pallidum* strain resistant to azithromycin. Nested PCR was performed to amplify the 23S ribosomal RNA (*23SrRNA*) gene. The point mutation of A2058G in *23SrRNA*, which confers *Treponema pallidum* resistance to azithromycin, was measured by endonuclease digestion of PCR amplification products using *Mbo*II. A2058G point mutation was detected in 91.0% (162/178; 95% CI, 86.8%, 95.2%) of the specimens, but no difference in prevalence of azithromycin resistance was found between the patients who had taken antibiotics before enrollment and the patients who had not (91.8% vs. 89.4%), nor between the patients with and without past sexually transmitted infections (87.1% vs. 93.1%). We concluded that azithromycin may not be suitable for syphilis as a treatment option in Guangxi Zhuang Autonomous Region because of the extremely high prevalence of resistance in the general syphilis population.

## Introduction

Syphilis, which is caused by *Treponema pallidum (T*. *pallidum)* and transmitted through sexual contact, remains a significant public health issue globally because of its damage to the body and vertical transmission. The majority of syphilis cases occur in developing countries, including China where the prevalence of syphilis increased from 6.1/100 000 people in 2001 to 32.0/100 000 in 2011, exceeding that of other countries[1]. Antibiotic treatment is one of the most important strategies for syphilis control. A single intramuscular injection of penicillin G benzathine is recommended as the first line treatment for early syphilis. Although no resistant cases were reported till now, the pain of injection of benzathine and the threat to patient’s life because of severe allergy to penicillin limit its application, and oral antibiotics including doxycycline and azithromycin are recommended as alternative treatments for syphilis in the UK and USA Centers for Disease Control and Prevention Guideline for the Diagnosis and Treatment of Syphilis[2,3]. To date, no *T*. *pallidum* strains resistant to tetracyclines, such as doxycycline, have been described, but the side effects including browning teeth and impaired bone development of children restrict their clinical usage, especially in pregnant women and children. At the beginning of the 21^st^ century, azithromycin was recommended as an alternative for patients who are allergic to penicillin[2,3] due to the advantage of a single dose oral administration, which has equivalent treatment effects to that of penicillin[4]. It is much more convenient for the subjects to deliver azithromycin to their sexual partners as prophylactic antibiotics usage, as well as for outreach workers to deliver it in population with high risk factors to increase treatment coverage, particularly outside the United States[5]. However, it is reported that the prevalence of azithromycin-resistant *T*. *pallidum* strains has been accompanied with treatment failure since 2002[6]. A gene mutation related to resistance was found and, consequently, a detection method was established[7]. Monitoring for azithromycin treatment failure and resistance of *T*. *pallidum* strains to macrolides has already drawn wide attention in China, for example, one hundred percent of azithromycin-resistant cases was reported in Shanghai[8] and 93.8% in Nanjing[9], China.

Guangxi Zhuang Autonomous Region, located in southwest China, geographically connects with Yuannan Province, Guizhou Province, Guangdong Province, and Vietnam. Because of the relatively low social-economic status and quality of medical care, Guangxi has faced serious difficulties of syphilis treatment and control in recent years[10]. In the present research, we aimed to investigate the prevalence of azithromycin-resistant *T*. *pallidum* strains and the epidemiological characteristics among patients with early syphilis in Guangxi Zhuang Autonomous Region, which may provide effective evidence to guide strategy makers for local disease prevention and control.

## Materials and Methods

### Subjects

This research was conducted from January 2012 to December 2014, and enrollment was performed in sexually transmitted infections (STIs) clinics in four cities (Nanning, Yulin, Liuzhou, and Fangchenggang) in Guangxi Zhuang Autonomous Region. Patients with typical syphilitic lesions such as genital ulcer of primary stage or skin lesions of secondary stage, and who were positive for rapid plasma reagin (RPR, described in section 3) and *T*. *pallidum* particle agglutination (TPPA, describe in section 4) were recruited.

All participants were interviewed *via* questionnaire with questions regarding their sociodemographic information, STIs and antibiotic exposure history, and sex behavior model (S1 Table). Serum RPR titer was recorded for further analysis. A routine physical examination, disease counseling, health education, and standard treatment were provided to all participants according to the national guidelines.

This study was approved by the Ethics Committee of the Dermatology Institute of Guangxi Autonomous Region, and written informed consent was obtained from all subjects prior to interview and sample collection.

### Samples collection

Venous blood samples were collected. Specimens were collected from condylomata lata, moist genital ulcers, papulae, or mucosal patches by swab. Crusts or necrotic materials were gently removed using sterile gauze before sampling. The lesions were scarified gently using a blunt scalpel to collect clear exudates with a sterile swab, and gently rub on the base of lesion before sampling. The swab was vigorously swirled with 1 mL of buffer (10 mmol/L Tris-HCl of pH 8.0, 0.1 mol/L EDTA of pH 8.0, and 0.5% SDS) for 15 seconds in a tube, and then pressed against the side to express the excess liquid. The tube was capped, labeled with a serial number, and temporarily stored at -20°C in the local laboratory.

### RPR card test

The Macro-Vue RPR Card Test (Becton Dickinson BD Microbiology Systems) uses cardiolipin antigen with a carbon particle to detect reagin. Reagin binds to the test antigen, which consists of cardiolipin–lecithin–cholesterol particles, causing macroscopic flocculation. Controls were established for each test to confirm optimal reactivity of the antigen. The test procedure followed the manufacturer’s instructions.

### *T*. *pallidum* particle agglutination (TPPA)

A 100 μL sample of diluent and 25 μL specimen were mixed, and then twofold serial dilutions were made with 25 μL diluent. The sensitized particles were serially mixed in the adjacent wells with a plate mixer for 30 seconds. After incubation at room temperature for 2 hours, the result of agglutination was read. The Serodia TPPA assay (Fujirebio, Tokyo, Japan) results were interpreted using the agglutination patterns of positive and negative controls.

### Genotyping

QIAamp DNA mini Kit (Qiagen, GmbH, Germany) was used for DNA extraction according to the manufacturer’s instructions and *arp*, *tpr*, and *tp0548* were amplified for gene typing using the primers in Table 1[11]. DNA extraction and PCR were performed at the Dermatology Institute of Guangxi Zhuang Autonomous Region.

**Table 1:** Primers used in the study.

Gene	Forward primer	Reverse primer
***arp***	5'-CAAGTCAGGACGGACTGTCC-3'	5'-GGTATCACCTGGGGATGC-3'
***tpr***	5'-CAGGTTTTGCCGTTAAGC-3'	5'-AATCAAGGGAGAATACCGTC-3'
***tp0548***	5'-GGTCCCTATGATATCGTGTTCG-3'	5'-GTCATGGATCTGCGAGTGG-3'

### Detection of azithromycin resistance

The *polA* gene was amplified using a method reported previously[12]. Nested PCR was used to amplify the 23S ribosomal *R*NA (*23SrRNA*) gene, and then the amplified product was digested with restriction enzyme *Mbo*II to detect the presence of the A2058G point mutation as described previously[13]. DNA extraction and PCR detection were performed at the Dermatology Institute of Guangxi Zhuang Autonomous Region.

### Statistical analysis

Two research assistants independently entered the questionnaire records and results into an EpiData database (version 3.1, The EpiData Association, Denmark), and congruency of database was evaluated. The percentage of positivity for syphilis and azithromycin resistant rate were calculated with 95% confidence intervals (CIs). χ^2^ test or the Fisher exact test was used to analyze the associations between categorical variables. The difference between continuous variables was determined by Student’s *t*-test. *P* values of <0.05 were considered to have statistical significance. All statistical analyses were performed using SPSS software (SPSS, version 15.0; SPSS Inc., Chicago, IL, USA).

## Results and Discussion

Two hundred and sixty-five patients were clinically suspected as syphilis, among which 183 were confirmed as *T*. *pallidum* infection by using Silver Stains Assay or serological tests including RPR (non treponema antibody test) and TPPA (specific antibody test of *T*. *pallidum*). Finally, a total of 178 patients with positive PCR amplification results were enrolled, consisting of 141 men (79.21%) and 37 women (20.79%). Among them, 177 received standard treatment for syphilis (benzathine penicillin G 2.4 million units intramuscularly once a week for 2 weeks), and one patient took azithromycin (1 g oral once).

The mean age of the subjects was 37.2 years (range from 17 to 85 years). Out of the 178 patients, 103 (57.87%) were married, 122 (68.0%) received high school or above education, and 82 (46.08%) were in a business occupation. About two thirds of the population were migrants (113, 63.5%), the majority of whom immigrated for more than 3 months rather than less than 3 months (67 vs. 46, 37.64% vs. 25.84%) (Table 2).

**Table 2:** Demographic characteristics of patients diagnosed with syphilis.

Demographic characteristics	Total number	Value (95%CI)
**Age, years**	178	37.2±13.96
**Male gender, n**	141	79.21 (73.25, 85.17)
**Married, n**	103	57.87 (50.63, 65.10)
**High school or above education**	122	68.0 (61.12, 74.83)
**Business Occupation**	82	46.08 (38.74,53.79)
**Immigrant resident for more than 3 months**	67	37.64 (30.52, 44.76)

CI: confidence interval.

Of the 178 subjects, 147(82.58%) purchased sex service, and 152 (85.39%) had commercial sex behavior, indicating that a small proportion of the subjects exchanged sex for drugs or other resources. None of the male subjects had anal sex with other men. In our study, most of the subjects (141, 79.21%) were men, and purchasing sex service was very common in this population, which significantly increased the infection risk of syphilis. The ratio of commercial sex behavior was much higher than that of another study reported in the same region[14]; the difference may be caused by the male/female ratio of subjects. Five persons (2.89%) were positive for HIV infection. Of all the subjects, 101 (56.7%, 95% CI, 49.5%-64.1%) were diagnosed as primary syphilis, compared to 77 secondary syphilis cases (43.3%, 95%CI, 35.5%-50.5%), and no significant difference was found between the lesions of patients with primary and secondary syphilis (χ2 = 1.468, *P* = 0.225). One hundred and four patients (58.4%) showed 2 or more clinical manifestations and 36% of the patients had 1:32 serum RPR titer at enrollment. Overall, 17 strain types were detected, and 14d/f was predominant (46.7%, 82⁄178, 95%CI, 38.7,53.4)(Table 3), which coincided with the findings in other areas in China and the USA[15].

**Table 3:** Sex behavioral and clinical and laboratory characteristics of patients diagnosed with syphilis (n = 178).

Characteristics	Total Number	Value(95%CI)
***Sex behavioral characteristics***		
**Purchase sex, n (% 95%)**	147	82.58 (77.01,88.15)
Commercial sex behavior *, n (% 95%)	152	85.39 (80.20, 90.58)
**Anal sex with other men**	0	NA
**HIV infection, n (%, 95% CI)**	5	2.89 (0.42, 5.35)
***Disease characteristics***		
**Primary stage**	101	56.7 (49.5,64.0)
**Median RPR titer (IQR)**	32	8–64
**14d/f Genotype of *Treponema pallidum***	82	46.7 (38.7,53.4)
**Resistance to azithromycin**	162	91.0 (86.8, 95.2)

*Commercial sex behavior: exchange sex behavior for things like money, drugs, or other resources, the subject could provide the sex service or receive the sex service. IQR: indicates interquartile range; RPR, rapid plasma reagin; NA, not available.

The three top types of *T*. *pallidum* distributing in Nanning are 14d/f (46.7%), 15d/f (13.4%) and 16d/f (11.2%), which highly corroborates the strain distribution previously reported in China[8,11], Rare types of 9h/c (2/178) and 15i/f (1/178) expect type j were also confirmed in our study, and all of the three strains are from migrant residents, which may explain the importation of these rare genotypes and suggest a potential linked transmission network in China.

Among the 178 PCR results, 91.0% (162/178; 95% CI, 86.8, 95.2%) contained the mutation of A2058G in *23SrRNA*. There is no difference in the prevalence of azithromycin resistance between the patients who took antibiotics and who did not (91.8% vs. 89.4%, OR = 1.321, *P*>0.05), nor between patients with and without STI history (87.1% vs. 93.1%, OR = 0.5, *P*>0.05) (Table 4). We also statistically analyzed the difference in resistance to azithromycin between 17 *T*. *pallidum* genotypes using Fisher test, and the results showed no difference, which may be affected by the number of samples; in parts of the types, the sample size is very small(<5).

**Table 4:** History of STIs and macrolide use by patients (n = 178).

Variable	Number	Resistance,%(95% CI)	OR	*P* value
**History of STIs**				
**Yes**	62	87.1%(82.2%,92.0%)	0.5	0.182
**No**	116	93.1%(89.4%,96.8%)	——	
**Previous use of macrolides**				
**Yes**	159	91.8%(87.8%,95.8%)	1.321	0.655
**No**	19	89.4%(85.0%,92.9%)	——	

STIs, sexually transmitted infections; CI, confidence interval; OR, odds ratios.

An important factor of antibiotic resistance in populations is antibiotic selection pressure. Previous exposure to macrolides was considered as a high risk factor of azithromycin resistance and, more persuasively, Chen *et al*.[9] reported that the relative risk of the A2058G mutation was 19.65 (*P*< 0.001) for patients who had a history of macrolide exposure, compared with those who had not. The resistance prevalence in China is extremely higher than that of other developing countries, which may be due to the fact that Chinese are taking 10 times more antibiotics on average compared with other nations[16]. The above theory is also supported by a high prevalence rate of azithromycin resistance to syphilis reported in other studies conducted in China, e.g. 100% in Shanghai[8] and 93.8% in Nanjing[9], while Taiwan has a much low azithromycin resistance rate (1.3% from 310 samples from patients with early syphilis) since macrolides are infrequently used in that location[17].

Another possible cause of high syphilis resistance to azithromycin in Guangxi may be the spread of azithromycin resistant *T*. *pallidum* strains. The genotyping results showed that the predominant strain types of *T*. *pallidum* distributions in Nanning are 14d/f (46.7%), 15d/f(13.4%) and 16d/f(11.2%), accounting for 71.3% of the entire samples, which completely corroborates strain distributions in China[11], and may explain the transmission networks in different areas of China.

The prevalence of azithromycin resistance in our study was 91.0%, which is consistent with the resistance rates (88.6%-95.2%) reported in other areas in China. However, further analyses indicated that there was no difference in the prevalence of azithromycin resistance between the patients who had taken antibiotics and who had not, which means exposure to macrolides may not be a risk factor for azithromycin resistance in syphilis in Guangxi. The prevalence of azithromycin resistance in patients without macrolides exposure may overwhelm the risk of being exposed to macrolides (89.4% in our study compared to 62.5% reported elsewhere[9]). The general high resistance of azithromycin to *T*. *pallidum* in the current study indicated that the situation for syphilis prevention and control is much more aggregate than that in 2012 in Guangxi, when azithromycin resistance in patients without exposure history was significantly lower, and azithromycin still worked in its study population[9].

Except *T*. *pallidum*, azithromycin resistance has been reported in *Neisseria gonorrhoeae* and *Mycoplasma genitalium*[17]. The molecular epidemiology showed that the main mutations related to macrolide resistance in *Mycoplasma genitalium* also located in nucleotide 2058 or 2059 in *23S rRNA*[18]. However, prolonged azithromycin treatment has been documented [19] because the 1 g single dose may be more likely to select for resistance compared with the extended 5 to 10 days regimen[20]. Each log10 increase of organism load was reported to be accompanied by a remarkable raise of the odds of 1 g single dose of treatment failure (adjusted OR is 1.8 with *P*<0.05)[21], so it is possible that a high beginning organism load of azithromycin leads to more organisms subsistence with the start peak concentrations, and then the surviving cells replicate when concentrations decrease below the minimal inhibitory concentration and the mutations would be readily selected, which may be one of the important causes of resistance of azithromycin to *T*. *pallidum* using 1 g single dose oral treatment.

Some limitations need to be addressed. Firstly, though this study contained numerous patients recruited from 4 cities to evaluate the prevalence rate of azithromycin resistance in Guangxi, the situations may be very different in other areas, particularly in rural and urban/rural integration areas, which may affect resistance detected. Secondly, the data regarding sexual behavior and history of macrolide administration was subject to self-reporting bias and recall bias. Third, a new mutation of A2059G mutation was firstly identified in 2009, and based on the recently reported studies, the mutations on A2058G and A2059G were not detected simultaneously[22], which means that actually resistance of azithromycin to *T*. *pallidum* may be higher than that reported, as the A2059 mutation has not been recommended by the Chinese Center for Disease Control and Prevention, so the detection has not been performed in the first line clinics in China. Till now, there is no report on *T*. *pallidum* resistant strains with simultaneous mutations at 2058 and 2059 position. A bigger size sample study should be conducted to include the two mutations (A2058G and A2059G) in the future, and further analyze the relationships between locations and genotypes.

## Conclusions

Based on the above results, azithromycin resistance is very common in the general syphilis population in Guangxi Zhuang Autonomous Region. This finding is regardless of STIs/history of STIs or macrolide administration history, which makes azithromycin unsuitable for syphilis treatment.

## Supporting Information

S1 Table Questionnaire for Patients Visiting STI Clinics. (DOC)
