# Supplementary material for: High Resistance to Azithromycin in Clinical Samples from Patients with Sexually Transmitted Diseases in Guangxi Zhuang Autonomous Region, China
Source: PLoS One. 2016 Jul 28;11(7):e0159787. doi: 10.1371/journal.pone.0159787 (PMC4965067; doi:10.1371/journal.pone.0159787)
Supplement: S1 Table — (DOC) [file pone.0159787.s001.doc]

**Questionnaire for Patients Visiting STI Clinics**

***Clinic：广西皮肤病防治研究所 Doctor's name：______________***

| 1.1 | | Date | 20|__||__|（year）|__||__|（month）|__||__|（day） | 请贴在此处 | |
| --- | --- | --- | --- | --- | --- |
| 1.2 | | Patient Number | |
| 1.3 | | Age | | | |__||__|（years old） |
| 1.4 | | Gander  **1=** male； **2=** female | | | |__| |
| 1.5 | | Marriage status  **1=** single； **2=** marriage； **3=** divorced； **4=** live together；**5=** widowed；**6=** remarriage；**7=** no response | | | |__| |
| 1.5 | | Education level)：  **1=** primary school and below； **2=** junior high school； **3=** high school/technical high school； **4=** junior college and above | | | |__| |
| 1.6 | | Occupation：  **1=**worker；**2=**farmer；**3=**cadre；**4=**service staff；**5=**teacher/technical personnel；**6**=rural migrant worker；**7=**individual household； **8=**unemployed； **9=**others (remarks**________________**） | | | |__| |
| 1.7 | | Location  1=resident；2=immigrant resident more than 3 months；3=immigrant resident less than 3 months；4=others（remarks：_______） | | | |__| |
| 1.8 | | Annual income)：  1= <5000 RMB； 2= 5000-10000 RMB； 3=10001-20000 RMB； 4=20001-30000 RMB；  5=30001-40000 RMB； 6= >40000； 7= no response | | | |__| |
|  | | | | | |
| 1.9 | Has syphilis infection history or not?  1= yes, go to 1.10； 2= no, please go to 1.12） | | | | |__| |
| 1.10 | Was treated for syphilis? Administration method  **1=** intragluteal injection；**2=** oral administration；**3=** intravenous administration；**4=** others（remarks**: ____________**） | | | | |__| |
| 1.11 | Treatment medicine  1= benzathine benzoylpenicillin； 2= procaine benzylpenicillin； 3= doxycycline； 4= tetracycline； 5=erythromycin；6= azithromycin; 7= ceftriaxone；8= others（remarks: ____________）；9= don't know | | | | |__| |
| 1.12 | Other STIs history  1= [Gonorrhoea](javascript:void(0);)；2= Chlamydial trachomatis；3= nongonococcal urethritis；4= [herpes](javascript:void(0);) [progenitalis](javascript:void(0);)；5= Condyloma acuminatum；  6= others（remarks：____________）；7= never | | | | |__| |
| 1.13 | Previously use of azithromycin?  1=yes；2= no, please go to 1.16；3= don't know，please go to 1.16 | | | | |__| |
| 1.14 | Aim of use of azithromycin?  （Treatment of **________________________________________**） | | | |  |
| 1.15 | Previously use of macrolides（for example: roxithromycin, clarithromycin）：  **1=**yes；**2=**no, please go to 1.1**9**；**3=** don't know, please go to 1.1**9** | | | | |__| |
| 1.16 | Previously use of Maddie fosfomycin, meleumycin, ethyl spiramycin, josamycin or kitasamycin ？  **1=**yes；**2=**no；**3=** don't know | | | | |__| |
|  | | | | | |
| 1.17 | | Did you provide sex service to others?  **1=**yes；**2=**no；**3=**no response | | | |__| |
| 1.18 | | Did you purchase sex service?  **1=**yes；**2=**no；**3=**no response | | | |__| |
| 1.19 | | Did you have anal sex with other men? (This question is only for male )  **1=**yes；**2=**no；**3=** no response | | | |__| |

**Only for doctors**

| 2.1 | Clinical manifestations ofsubjects enrolled（Multiple-choice）：  **1=**genital ulcer ；**2=** hard chancre；**3=** swollen inguinal lymph nodes；**4=** condylomata lata；**5=** palmoplantar typhus；**6=** urethra / vaginal discharge；**7=** genital vegetation；**8=** blister | |__||__||__||__||__| |
| --- | --- | --- |
| 2.2 | Other clinical manifestations：**________________________________________________________** |  |
| 2.3 | Did the subjects take dark field examination of syphilis  **1=** yes；**2=**no, please go to **2.5** | |__| |
| 2.4 | Result of dark field examination of syphilis  **1=**positive；**2=**negative | |__| |
| 2.5 | Non-specificity detection method for syphilis  **1=**RPR；**2=**TRUST；**3=**others（remarks：**____________**） | |__| |
| 2.6 | Result of RPR or TRUST  **1=Postive （please provide the titer); 2=Negative (**0:000**）** | |__|:|__||__||__| |
| 2.7 | Specificity detection method for syphilis  1=**TPPA**; 2=**TPHA；**3=**Rapid test；**4= **others（remarks：____________）** | |__| |
| 2.8 | Result of TPPA、TPHA or Rapid test  **1=** postive；**2=** negative | |__| |
| 2.9 | Diagnosis of the subject  **1=** [primary stage of syphilis](http://www.baidu.com/link?url=3JKB6kkVtjOQZ_kmcNKUBTE33b7rjsVdY3jf_56a4ItMxYpDkzlwdB_8D6VTKIHMhxU0TRDp8_9UfsYgPNpFsS4bFbxf3eVHhAmn1oHs4sRA_oT08hHd9YCf-iU8K1Oe)；**2=** tertiary syphilis；**4=**others（remarks**____________**） |  |
| 2.10 | Treatment  Medicine：**________________**；Dose：**________________**；Course：**________________** |  |
| 2.11.1  2.11.2  2.11.3  2.11.4  2.11.5 | The results and methods for other STI pathogen detection  Method **1=**（**____________**） Result**1=**（**____________**）  Method **2=**（**____________**） Result **2=**（**____________**）  Method **3=**（**____________**） Result **3=**（**____________**）  Method **4=**（**____________**） Result **4=**（**____________**）  Method **5=**（**____________**） Result **5=**（**____________**） | |__| |
| 2.12 | Did the subject take HIVtest？  **1=**yes； **2=**no | |__| |
| 2.13 | HIV result  **1=** postive；**2=** negative | |__| |
